# Parental Refusal to Lumbar Puncture: Effects on Treatment, Hospital Stay and Leave Against Medical Advice

**DOI:** 10.7759/cureus.7781

**Published:** 2020-04-22

**Authors:** Mushtaq Ahmed, Muzamil Ejaz, Saad Nasir, Salma Mainosh, Ashraf Jahangeer, Mahnoor Bhatty, Zobia Razi

**Affiliations:** 1 Pediatrics, Civil Hospital Karachi, Dow University of Health Sciences, Karachi, PAK; 2 Internal Medicine, United Medical and Dental College, Creek General Hospital, Karachi, PAK; 3 Epidemiology, Civil Hospital Karachi, Dow University of Health Sciences, Karachi, PAK; 4 Pediatrics, Dow Medical College, Karachi, PAK

**Keywords:** lumbar puncture refusal, cns infections, leave against medical advice, meningitis

## Abstract

Introduction

Lumbar puncture (LP) is an effective method in the diagnosis and management of central nervous system infections. Refusal to LP is associated with severe consequences. This study aims to examine the impact of parental LP refusal on treatment, the length of hospital stay, and the frequency of patients leaving against medical advice (LAMA).

Method

A cross-sectional study was conducted at the pediatric department of Civil Hospital, Karachi, from June 2018 to November 2019. All hospitalized patients suspected to have a central nervous system disease, which requires LP, were enrolled. Patients were followed for the duration of antibiotic and antiviral therapy, length of hospital stay, and LAMA.

Results

A total of 220 patients participated in the study, with the median age of nine (2-47) months. There were 113 (51.1%) males. The median length of hospital stay was 10 (4-14) days. The comparison of parental LP refusal with the length of hospital stay showed a significant difference (p-value <0.001) in the number of days of treatment among patients who received vancomycin (p-value =0.008) and meropenem (p-value =0.012). A significant association of parental LP refusal was also observed with meningoencephalitis and meningitis as provisional diagnosis (p-value =0.006). In particular, LAMA and death were found significantly higher among parents who refused LP (p-value <0.001).

Conclusion

LP refusal has a significant effect on the treatment, hospital stay, and disposition outcomes. A large number of parents who declined the procedure left against medical advice or suffered grave medical consequences. Parental education addressing their concerns and beliefs, while explaining the indications, and need for performing LP can help effectively overcome this issue.

## Introduction

Lumbar puncture (LP) is an important procedure for the diagnosis and management of central nervous system (CNS) infections in children and adults, and refusal to this procedure is an issue that is encountered worldwide with a refusal rate as low as 5% in the United States to 24.7% in Malaysia, and even much higher refusal rates are observed in Iran and Kuwait of about 62% and 80%, respectively [1,2]. The refusal rate varies with the cultural beliefs of a population and their knowledge about the procedure [1-4]. A study by Ahmad et al. including 215 patients with indications for LP, of which 70 (32.6%) families refused LP for their children, revealed that the most common reason for the parental refusal to LP was fear of limb paralysis (64.2%), followed by fear of death (31.3%), while in 19.4% of the cases, the parents considered the procedure unnecessary [4].

LP is a frequently performed procedure in pediatrics. This is particularly important in newborns and children less than 12 months of age, where, according to the American Academy of Pediatrics, LP should be performed to rule out meningitis, which may present asymptomatically or with masked symptoms [1]. In the case of meningitis, LP refusal may lead to frequent hospital admissions for empiric antibiotic therapy and increased duration of hospital stay, which may further expose patients to nosocomial infections [3,5].

Failure to perform LP in resource-limited settings is associated with greater morbidity and mortality secondary to delayed diagnosis and improper treatment [5]. A prospective study by Narchi et al. included 55 families, of which 24 (44%) refused LP, while awareness of complications (such as seizures, deafness, and blindness) associated with meningitis was a significant factor for parents consenting to the procedure [6]. Refusal to LP is associated with extended duration of antibiotic therapy, prolonged hospital stay, and a higher likelihood of leaving against medical advice (LAMA) causing additional burden to the health care system [5-7].

Studies on LP and its implications on the management and health care system are still lacking in developing countries; hence, the purpose of this study is to evaluate the outcomes of parental refusal to LP on treatment, length of hospital stay, and frequency of LAMA.

## Materials and methods

This cross-sectional study was conducted at the pediatric department of Civil Hospital, Karachi, from June 2018 to November 2019. All patients suspected to have a CNS disease, requiring an LP, were enrolled. All patients referred from other hospitals with LP done before admission in Civil Hospital or patients referred from Civil Hospital to any other hospital for the need for ventilator support were excluded.

LP is the process of obtaining cerebrospinal fluid from the lower part of the spine using a needle for the diagnosis of suspected CNS infections. Disposition outcomes such as discharge well, discharge on request, LAMA, and death were noted. The act of leaving the hospital without informing doctor/hospital administration or discharge/ leave from the hospital after signing a declaration that clearly states that discharge is contrary to the advice of a medical team of management was labeled as LAMA.

Patients were divided into two groups based on the LP procedure status. Those who underwent LP and those with a parental refusal to LP. Data were collected from the hospital admission record through a questionnaire. Age, gender, diagnosis, LP status, antibiotic, and antiviral therapy initiation were recorded from the medical record. Patients were followed for the duration of antibiotic and antiviral therapy, the length of hospital stay, and LAMA.

Data were entered into and analyzed using Statistical Package for Social Science, version 2 (IBM Corp., Armonk, NY). Descriptive statistics like median and interquartile range were calculated for age, and frequency distribution was calculated for gender, diagnosis, and LP status. Chi-square test and Mann-Whitney U test were applied to calculate the p-value for comparison of LP status with the duration of therapy, length of hospital stays, and LAMA. A p-value of less than 0.05 was considered statistically significant.

## Results

A total of 220 patients participated in the study, of whom 113 (51.1%) were males. The median age of the patients was nine (2-47) months, and there were 108 (48.9%) females. The median length of stay was 10 (4-14) days.

LP was performed in 110 patients, an equal number of patients with parental refusal also included. The provisional diagnosis showed that meningitis and meningoencephalitis were found in a higher number of patients, i.e. 120 (54.3%) and 56 (25.3%), respectively.

The comparison of LP refusal with general characteristics of the patients showed a significant median difference in length of hospital stay with LP refusal (p-value <0.001).

The frequency of antibiotic therapy showed that vancomycin was administered most frequently (198; 89.5%), followed by ceftriaxone (120; 54.2%), cefotaxime (79; 35.7%), and meropenem (62; 28%), while a small number of patients also received amikacin (30; 13.5%), and piperacillin-tazobactam (18; 8.1%). Antiviral therapy (acyclovir) was administered to 98 (44.3%) patients. The median number of days antibiotic therapy was given is mentioned in detail in Table 1. Moreover, the median difference between the number of days of treatment showed a significant difference among patients who received vancomycin (p-value =0.008) and meropenem (p-value =0.012). Similarly, a provisional diagnosis of meningitis and meningoencephalitis (p-value =0.006) was also found to be significantly associated with LP refusal (Table 1).

**Table 1:** Comparison of lumbar puncture performed with general characteristics of the patients (n=220) ‡Mann-Whitney U test applied. ϮChi-square test applied. *P-value <0.05. n, sample size; IQR, interquartile range.

	Total	Lumbar Puncture		P-Value
		Done (n=110)	Refused (n=110)	
	Median (IQR)	Median (IQR)	Median (IQR)	
Age, months	9 (2 - 47)	9 (1-36)	10 (2-60)	0.295^‡^
Length of hospital stay, days	10 (4-14)	11 (7-15)	8 (2-13)	<0.001^‡*^
Duration of antibiotics, days				
Ceftriaxone (n=120)	5 (2-10)	7 (3-11)	4 (1-10)	0.176^‡^
Vancomycin (n=198)	8 (4-12)	9 (6-13)	7 (2-11)	0.008^‡*^
Acyclovir (n=98)	8 (5-12)	8 (5-12)	9 (3-11)	0.657^‡^
Cefotaxime (n=79)	8 (4-11)	7 (3-10)	9 (4-11)	0.262^‡^
Meropenem (n=62)	8 (4-12)	8 (6-14)	5 (1-10)	0.012^‡*^
Piperacillin-tazobactam (n=18)	5 (1-10)	9 (1-12)	5 (2-5)	0.184^‡^
Amikacin (n=30)	3 (1-7)	3 (1-8)	2 (1-5)	0.512^‡^
Artesunate (n=13)	3 (2-5)	3 (3-5)	3 (1-3)	0.762^‡^
	n	n (%)	n (%)	
Gender				
Male	113 (51.1)	49 (43.4)	64 (56.6)	0.037^Ϯ*^
Female	108 (48.9)	62 (57.4)	46 (42.6)	
Provisional diagnosis				
Meningitis	120 (54.3)	55 (45.8)	65 (54.2)	0.006^ Ϯ*^
Encephalitis	11 (5)	8 (72.7)	3 (27.3)	
Meningoencephalitis	56 (25.3)	23 (41.1)	33 (58.9)	
Other	34 (15.4)	25 (73.5)	9 (26.5)	

**Figure 1:**
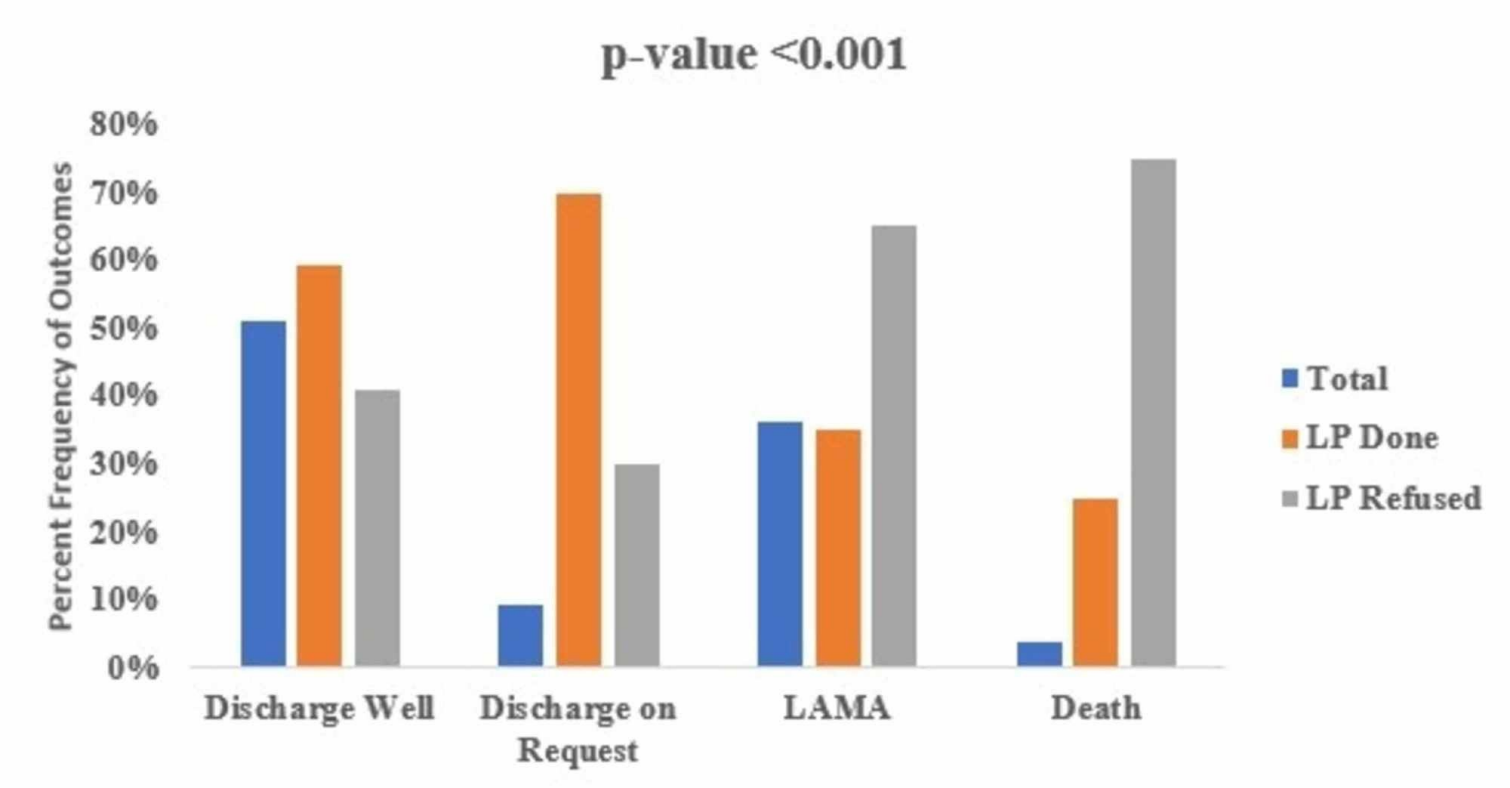
Comparison of refusal to LP with discharge status (n=221) n, sample size; LP, lumbar puncture; LAMA, leave against medical advice.

Analysis of the outcomes revealed that 113 (51.1%) patients discharged well, followed by 80 (36.2%) LAMA patients, 20 patients (9%) discharged on request, and eight (3.6%) patients died. LAMA and death were found significantly higher among patients with the parental refusal to LP (p-value <0.001) (Figure 1).

## Discussion

This study was done to evaluate the outcomes of refusal to LP on treatment, hospital stay, and LAMA patients. For this purpose, a comparison was done among patients with and without LP refusal by parents of children with a suspected CNS examination. The overall provisional diagnosis revealed that cases of meningitis were predominantly higher (54.3%), followed by meningoencephalitis (25.3%), and encephalitis (5%) in patients with a parental refusal to LP. In particular, the LP refusal rate was considerably higher among patients with meningoencephalitis, followed by meningitis (p-value =0.006).

Mijovic and Sadarangani proposed that even though newer diagnostic tests have improved the ability to detect the causal organism in cases such as bacterial meningitis, there remains a high reliance on clinical suspicion and LP can efficiently confirm the diagnosis without any delay in the absence of contraindications, and before the administration of antibiotics [8]. A study by Kneen et al. included children in whom a CNS infection was suspected, and reported that only 53% of the children, who had no contraindication to LP, underwent LP, while LP contributed in the management of 72% of these patients due to correct identification of the causing organism [9].

According to our study findings, the length of hospital stay was considerably higher among patients in whom LP was performed than that of patients who refused LP, although, when patients were compared based on disposition outcomes, it was observed that LAMA and death were significantly higher in patients with LP refusal, which explains the shorter duration of hospital stay in patients with a parental refusal to LP.

Several other studies support our findings [1-4]. Our literature search revealed that parental LP refusal is significantly associated with LAMA. Ling and Boey in their study revealed that parents who refused LP were significantly more likely to discharge their children against the medical advice (21% of refusal associated with LAMA vs only 3% of patients whose parents consented to LP associated with LAMA (p-value=0.04)) [7]. Another study by Sharif et al. reported a similar finding, where 55 families refused LP, of which 32 families also left against medical advice (58% LAMA rate with LP refusal) [1]. These patients who discharge against advice following parental refusal to LP are a great concern because diagnosis and management of serious conditions such meningitis might be further delayed, and could lead to grave consequences including death [7,10,11]. LAMA is associated with a higher risk of death, complications, and readmissions, as proposed by studies done by Serem et al. and Glasgow et al., which revealed a statistically significant association between LAMA and 30-day mortality and readmission rate [12,13].

Our findings suggest that amongst all enrolled patients, half of the patients discharged healthy from the hospital (51.1%) followed by LAMA (36.2%) and discharge on request (9%). While death was reported in eight patients. The patients in whom LP was performed were more likely to discharge well from the hospital. 

Patient management most frequently involved administration of vancomycin (89.59%), followed by ceftriaxone (54.29%), whereas piperacillin-tazobactam (8.15%) and amikacin (13.57%) were the least frequently administered antibiotics. The number of days of treatment showed that a significant median difference was observed in patients who received vancomycin and meropenem. The number of days of treatment was significantly higher among patients in whom LP was performed, which most likely increased the overall duration of hospital stay of these patients (as LAMA and discharge on request rates were low in patients who underwent LP), and they completed their therapy, which could explain the results. LP enables the examination of cerebrospinal fluid, which is often necessary for the diagnosis of CNS infections in the pediatric population. Despite its usefulness as a diagnostic technique, parental reluctance to agree to LP has been the subject of ongoing debate in various parts of the world, reflecting the universal nature of the issue [7,14-17].

Hence, LP refusal has a greater impact on the treatment, hospital stay, and decision of LAMA. Furthermore, our findings also emphasize the need for effective counseling of the parents/guardians to counter this issue. In a study by Dunbar et al., a decline was observed in the LP refusal by parents after counseling through educational video [14]. Several studies report that the cultural beliefs and myths in society may lead to increased refusal to LP by parents [15-18]. A study by Khakshour et al. reported that fear of complications from the procedure was the main reason for parental refusal, most common being fear of paralysis and low back pain, which leads to dissatisfaction in more than half of the parents [19].

This study reveals the current severity of the problem often faced by pediatricians in treating children with a CNS infection requiring LP. We believe that through our study, we further strengthen the existing literature by proposing that the refusal of this procedure by parents in children can lead to severe outcomes. An extensive prospective study, especially in developing countries, is suggested to consider other variables for parental refusal to LP and its implications. 

## Conclusions

Parental refusal to LP in children presumed to have CNS infections has a significant effect on the outcomes such as the length of hospital stay, the median difference between the number of days of treatment, and the frequency of LAMA. It is associated with a significantly higher number of deaths observed. We believe that educating the parents about the importance while acknowledging their previous perceptions, concerns, and cultural beliefs regarding the procedure can play a pivotal role to ensure prompt medical care and prevent serious consequences.
